# Effect of Recurrent Salt and Drought Stress Treatments on the Endangered Halophyte *Limonium angustebracteatum* Erben

**DOI:** 10.3390/plants12010191

**Published:** 2023-01-03

**Authors:** Roberta Calone, Diana-Maria Mircea, Sara González-Orenga, Monica Boscaiu, Javier Zuzunaga-Rosas, Lorenzo Barbanti, Oscar Vicente

**Affiliations:** 1 CREA—Council for Agricultural Research and Economics, Research Centre for Agriculture and Environment, I-40128 Bologna, I-00184 Rome, Italy; 2 Institute for Conservation and Improvement of Valencian Agrodiversity (COMAV), Universitat Politècnica de València, Camino de Vera 14, 46022 Valencia, Spain; 3 Faculty of Horticulture, University of Agricultural Sciences and Veterinary Medicine of Cluj-Napoca, 3-5 Manastur St., 400372 Cluj-Napoca, Romania; 4 Department of Plant Biology and Soil Science, Universidad de Vigo, Campus Lagoas-Marcosende, 36310 Vigo, Spain; 5 Mediterranean Agroforestry Institute (IAM), Universitat Politècnica de València, Camino de Vera 14, 46022 Valencia, Spain; 6 Department of Agricultural and Food Sciences, Alma Mater Studiorum, University of Bologna, Viale Fanin 44, 40127 Bologna, Italy

**Keywords:** recretohalophytes, salt stress, drought stress, stress recovery, stress memory, osmolytes, ion transport, oxidative stress markers, antioxidant metabolites

## Abstract

*Limonium angustebracteatum* is an endemic halophyte from the Spanish Mediterranean coastal salt marshes. To investigate this species’ ability to cope with recurrent drought and salt stress, one-year-old plants were subjected to two salt stress treatments (watering with 0.5 and 1 M NaCl solutions), one water stress treatment (complete irrigation withholding), or watered with non-saline water for the control, across three phases: first stress (30 days), recovery from both stresses (15 days), and second stress (15 days). Growth and biochemical parameters were determined after each period. The plants showed high salt tolerance but were sensitive to water deficit, as shown by the decrease in leaf fresh weight and water content, root water content, and photosynthetic pigments levels in response to the first water stress; then, they were restored to the respective control values upon recovery. Salt tolerance was partly based on the accumulation of Na^+^, Cl^−^ and Ca^2+^ in the roots and predominantly in the leaves; ion levels also decreased to control values during recovery. Organic osmolytes (proline and total soluble sugars), oxidative stress markers (malondialdehyde and H_2_O_2_), and antioxidant compounds (total phenolic compounds and flavonoids) increased by various degrees under the first salt and water stress treatments, and declined after recovery. The analysed variables increased again, but generally to a lesser extent, during the second stress phase, suggesting the occurrence of stress acclimation acquired by the activation of defence mechanisms during the first stress period.

## 1. Introduction

Coastal salt marshes are transition zones between land and sea that have expanded widely on low-energy coasts at intermediate and high latitudes, and are characterised by breaking water vegetation [1]. In the past, these habitats were considered insalubrious and of little benefit and they were, therefore, intensely converted to nontidal land for agriculture, urban development, or industrialisation [2]. Additionally, many of these areas suffer mounting touristic pressure, further contributing to their deterioration [3]. Today, many protection measures are in place to safeguard the remaining littoral salt marshes from human activity and the threat posed by the predicted rise in sea level as a consequence of climate change [4,5]. Indeed, nowadays, it is widely recognised that salt marshes perform highly valuable ecosystem services in coastal defence and wildlife conservation [6,7], providing a habitat for migratory waterfowl, transient marine species, and indigenous flora and fauna. Furthermore, it is acknowledged that they exert a major role in offsetting human greenhouse gas emissions by sequestering a considerable amount of carbon in vegetation and as peat [8]; indeed, it is estimated that about 430 × 10^6^ tonnes of C are stored in the upper 0.5 m of soils in salt marshes globally [9].

Salt marshes are colonised by distinctive halophytic vegetation, which includes an abundant population of structural dominant plant species together with rare, endemic, and/or endangered species of high ecological value [10]. According to their degree of salt tolerance, these halophytic plants are distributed following the salt marsh salinity gradient [11]. However, in Mediterranean salt marshes, soil salinity is not the only stressor flora has to contend with. Here, these plants must be able to switch from conditions of heavy rains and waterlogging during the spring season, when salts are leached to the deepest soil horizons, to extreme drought and high temperatures in summer, when salts accumulate on the soil surface due to the intense evapotranspiration [12,13].

*Limonium* Mill. (sea lavender; Plumbaginaceae) is a large halophytic genus including ca. 600 species [14] distributed worldwide [15]. They show remarkable variability in the chromosome number, ranging from 12 to 18 in diploids and from 24 to 72 in polyploids [15], and can reproduce both sexually and asexually via apomixis [16]. Many species of this genus are sympatric, coexisting within heterogeneous environments covering separate ecological niches as the result of adaptive phenotypic differentiation to varying environmental conditions [17].

As typical recretohalophytes, *Limonium* species possess peculiar morpho-anatomical structures responsible for salt excretion, i.e., salt bladders and salt glands, which are absent in glycophytes [18,19]. Furthermore, their salt tolerance is based on the presence of constitutive stress tolerance mechanisms, such as the active accumulation of mono and divalent ions in the leaves [20,21,22] and the high production of stress-induced osmolytes (especially proline and fructose) for osmotic adjustment, together with enhanced activity of antioxidant enzymes and the synthesis of antioxidant compounds [23,24]. Altogether, these mechanisms also confer fair drought resistance [25].

The Valencian Community on the eastern (i.e., Mediterranean) coast of Spain is one of the most biodiverse European regions in *Limonium*, sheltering 29 species, many of them endemic [26]. Amongst these, *Limonium angustebracteatum* Erben [27] is a C3 perennial typical of the clayey-sandy salt marshes of the eastern and southeastern Iberian coast (Alicante, Almeria, Castellon, Murcia, and Valencia provinces). According to Moreno et al. [28], who compared and classified different perennial *Limonium* species based on their morphological traits and plant-soil relationships, *L. angustebracteatum* belongs to the *Limonium* morphotype C. This *Limonium* group is characterised by the absence of sterile branches and the presence of green leaves, mostly coriaceous, at anthesis. It generally occurs in soils with high salinity (EC), Na^+^, K^+^, Cl^−^, and exchangeable sodium percentage (ESP), occupying an intermediate ecological position between succulent plant populations and *Lygeum spartum* steppes.

To better assess this plant’s ability to cope with environmental stress, this study aimed to characterise the responses of *L. angustebracteatum* after exposure to two periods of high salinity and drought stress, interspersed with a recovery period. Several studies indicate that after the first exposure to abiotic stress, plants may become more resistant [29] or more susceptible to subsequent stress events [30]. This variation in response to a specific stressor may be regarded as the ‘stress memory’ of plants [31], consisting of genetic, structural, and biochemical modifications induced by a first or recurrent exposure to stress, which will influence their resilience to stressful events [32]. Understanding these responses is becoming particularly relevant due to the sharpening of fluctuating environmental conditions caused by global warming. In this study, we have analysed the responses at the end of an initial stress, a recovery period, and a second stress in the endemic *L. angustebracteatum*, in terms of growth and concentration of specific biochemical stress markers (photosynthetic pigments, different mono- and divalent ions and organic osmolytes, oxidative stress markers, and antioxidant compounds). Information in this domain is considered of major importance to better understand the physiological plasticity that allows this halophyte to survive the extreme variability in soil salinity and water content that characterises Mediterranean coastal environments.

## 2. Results

### 2.1. Substrate Salinity and Water Content

**Figure 1:**
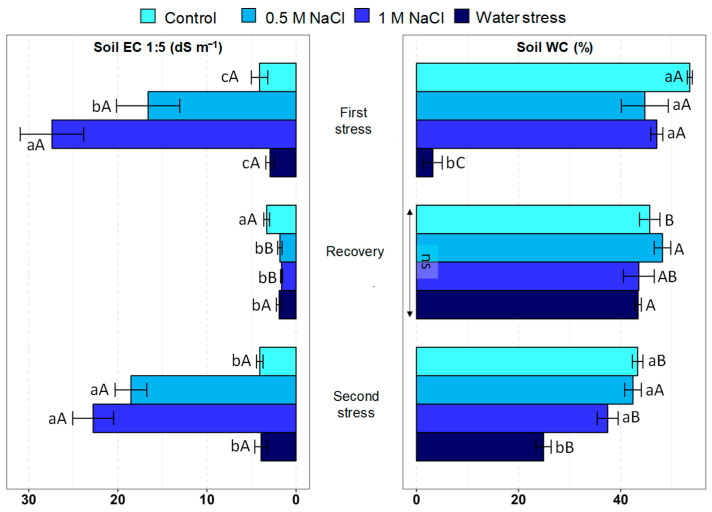
Effect of 30 days of stress treatment (First stress), followed by 15 days of watering with non-saline water (Recovery) and by additional 15 days of stress (Second stress) on substrate electrical conductivity (Soil EC 1:5, **left**) and water content (Soil WC, **right**). Different lowercase letters beside the bars indicate significant differences between treatments (Control, 0.5 M NaCl, 1 M NaCl, and Water stress) for each sampling, at *p* ≤ 0.05; ns: non-significant. Different uppercase letters indicate significant differences between the three sampling times (First stress, Recovery, and Second stress) for each treatment, at *p* ≤ 0.05; ns: non-significant. Vertical bars indicate standard error (n = 4).

Substrate salinity in terms of electrical conductivity (EC_1:5_) varied widely between experimental treatments and across the stress and recovery phases (Figure 1). As expected, no significant changes were detected in the (low) EC_1:5_ values measured in the pots of control and drought-treated plants after the three experimental phases—first stress, recovery, and second stress. Regarding the substrates of salt-treated plants, the first and second stress depicted similar patterns: a progressive increase in soil EC_1:5_ with increasing salinity (0.5 and 1 M NaCl), reaching a maximum value of over 25 dS m^−1^. During the intermediate recovery phase, soil EC_1:5_ in the two treatments involving previous NaCl addition (0.5 and 1 M NaCl) was restored to very low levels, even significantly lower than in the control.

Soil water content after the first stress treatment was upheld at the highest values (ca. 48%), without statistically significant differences between the control and the two salt concentrations, whereas it fell to very low levels (<5%) in the water stress treatment. After the recovery, soil water content was maintained at the same high level in all treatments; that is, the substrate of pots with plants subjected to water stress recovered the same moisture level as in the control and the salt-treated pots. Lastly, after the second stress period, the soil water content in the water stress treatment, about 25%, was significantly lower than in the control and salt treatments, although higher than after the first stress period (Figure 1). These data show that the recovery treatment was fully effective in restoring the control conditions, in terms of substrate electrical conductivity and moisture.

### 2.2. Growth Parameters

The experimental treatments across stress and recovery phases significantly influenced the total number of leaves and the proportion of senescing (i.e., wilted) leaves (Table 1). In the first stress period, the total leaf number was not significantly different between treatments, but the proportion of senescing leaves rose from 13% in the control to an average of 40% in the two strongest stress treatments (1 M NaCl and water stress). During the recovery phase, a complete recovery in the total leaf number and the proportion of senescing leaves was observed in plants subjected to the lower salinity treatment compared to the control. Conversely, plants subjected to the higher salinity treatment and to water stress showed a higher proportion of senescing leaves after recovery. After the second stress period, the overall effect of the first stress treatment, subsequent recovery, and repeated stress resulted in non-significant differences between treatments in leaf number or the proportion of senescing leaves.

Specific leaf area, which was not assessed at the beginning of the experiment, did not exhibit significant variations between treatments or when comparing the three samplings (Table 1). It appears, therefore, that the increase in the percentage of wilted leaves was not reflected in SLA differences during the experiment.

**Figure 2:**
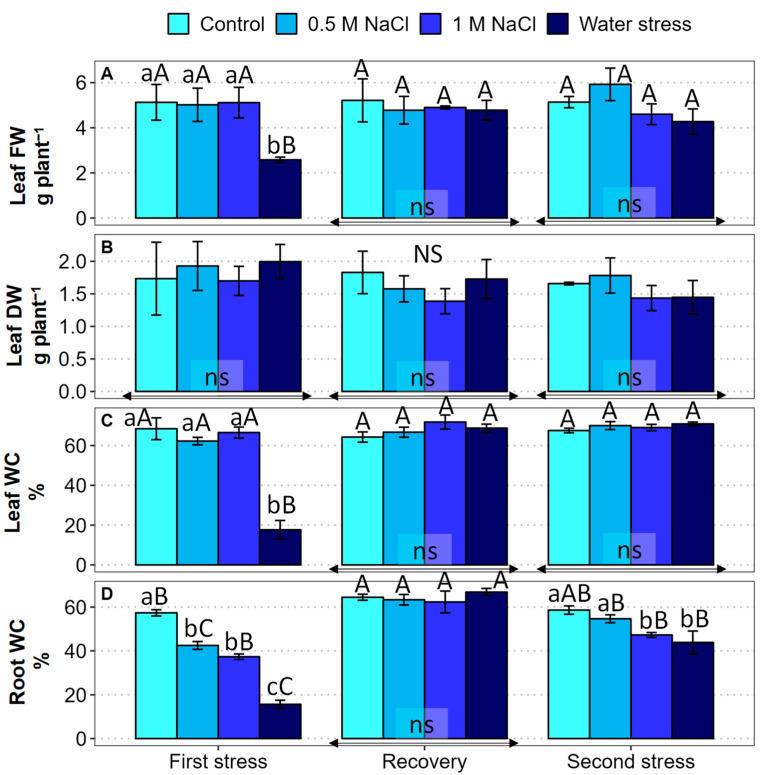
Effect of 30 days of stress treatments (First stress), followed by 15 days of watering with non-saline water (Recovery) and by additional 15 days of stress (Second stress) on (**A**) leaf fresh weight (Leaf FW), (**B**) leaf dry weight (Leaf DW), (**C**) leaf water content (Leaf WC), and (**D**) root water content (Root WC) of *Limonium angustebracteatum* plants. Different lowercase letters over the bars indicate significant differences between treatments (Control, 0.5 M NaCl, 1 M NaCl, and Water stress) for each sampling, at *p* ≤ 0.05; ns: non-significant. Different uppercase letters indicate significant differences between the three sampling times (First stress, Recovery, and Second stress) for each treatment, at *p* ≤ 0.05; ns: non-significant. Vertical bars indicate standard error (n = 4).

Leaf fresh weight (FW) and dry weight (DW), and water content (WC) of the leaves and roots were determined after the three experimental phases in control and stressed plants (Figure 2). After the first stress period, leaf FW did not vary in the presence of salt but dropped by more than 50% in the water stress treatment (Figure 2A). On the contrary, the first water and salt stress treatments did not cause significant changes in leaf DW compared to the non-stressed controls (Figure 2B). In the subsequent phases (recovery and second stress), no significant differences were detected between treatments in leaf FW or DW (Figure 2A,B). Similar to leaf FW, leaf WC did not vary in the control and the salt-treated plants throughout the three phases of the experiment but decreased below 20% after the first water stress period. Leaf WC was completely restored to control levels after watering with non-saline water and was not affected by the second water stress treatment (Figure 2C). This means that the drop in leaf FW observed after the first water stress treatment was primarily due to dehydration of the leaves and not to growth inhibition, i.e., reduction in biomass accumulation.

During the first stress phase, root water content (RWC) exhibited a significant decrease in the presence of salt, and a stronger reduction, to less than 20%, in plants subjected to water deficit (Figure 2D). After recovery, RWC was restored to high levels in all treatments. Responses to the second stress phase were milder than those observed after the first period; RWC decreased significantly in response to the 1 M NaCl and water stress treatments, but they were down to values over 40% in both cases (Figure 2D).

### 2.3. Photosynthetically Active Pigments

**Figure 3:**
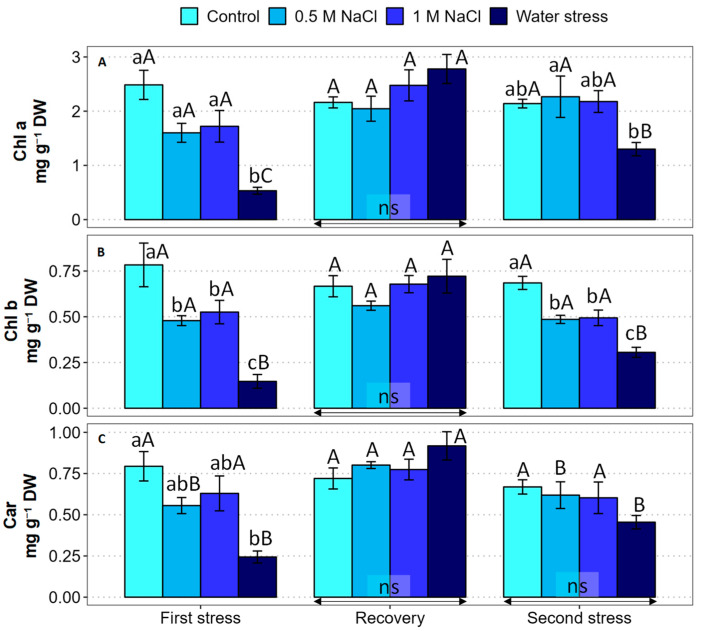
Effect of 30 days of stress treatment (First stress), followed by 15 days of watering with non-saline water (Recovery) and by additional 15 days of stress (Second stress) on the contents of photosynthetic pigments in *Limonium angustebracteatum* leaves. (**A**) Chlorophyll a (Chl a), (**B**) chlorophyll b (Chl b), and (**C**) total carotenoids (Car). Different lowercase letters over the bars indicate significant differences between treatments (Control, 0.5 M NaCl, 1 M NaCl, and Water stress) for each sampling at *p* ≤ 0.05; ns: non-significant. Different uppercase letters indicate significant differences between the three sampling times (First stress, Recovery, and Second stress) for each treatment, at *p* ≤ 0.05; ns: non-significant. Vertical bars indicate standard error (n = 4).

The content of photosynthetically active pigments showed some changes determined by the experimental treatments and phases (Figure 3). During the first stress period, average chlorophyll a (Chl a) levels were slightly reduced in salt-treated plants, with respect to the non-stressed control, but the differences were not statistically significant; on the contrary, a strong reduction was detected under water stress (Figure 3A). A similar qualitative variation pattern was observed for chlorophyll b (Chl b) contents: a slight (although, in this case, significant) reduction in response to the 0.5 and 1.0 M NaCl treatments and a much stronger decrease in the water-stressed plants (Figure 3B). Total carotenoid (Car) levels also decreased significantly in response to water deficit stress (Figure 3C). Watering with non-saline water led to a complete recovery, to control levels, of the three pigments in all the plants, without differences between treatments (Figure 3). The second stress treatments caused a similar but milder response than the first ones. No significant changes in Chl a and Car contents, and a slight reduction in Chl b, were observed in the presence of 0.5 and 1.0 M NaCl; on the other hand, a reduction in the average levels of the three pigments was measured in the water-stressed plants, although differences with the control were non-significant for Car contents (Figure 3).

### 2.4. Ion Accumulation

**Figure 4:**
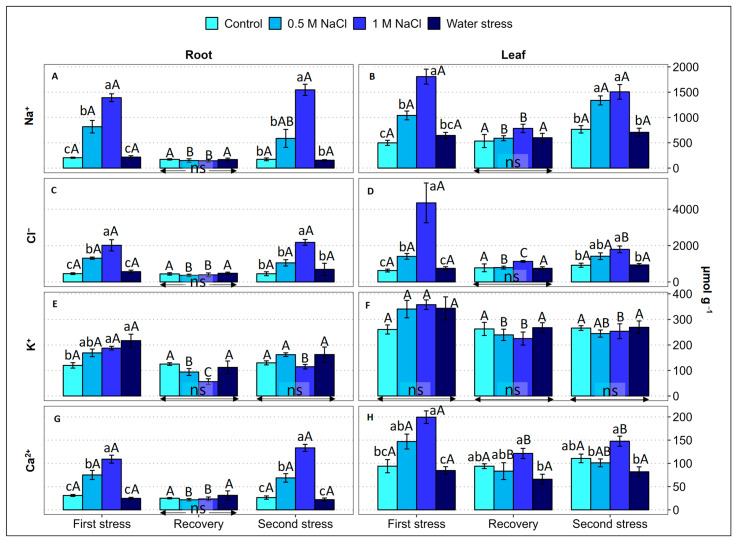
Effect of 30 days of stress treatment (First stress), followed by 15 days of watering with non-saline water (Recovery) and by additional 15 days of stress (Second stress) on the root and leaf concentration (in μmol g^−1^ DW) of ions, in *Limonium angustebracteatum* plants: (**A**,**B**) sodium (Na^+^), (**C**,**D**) chloride (Cl^−^), (**E**,**F**) potassium (K^+^) and (**G**,**H**) calcium (Ca^2+^). Different lowercase letters over the bars indicate significant differences between treatments (Control, 0.5 M NaCl, 1 M NaCl, and Water stress) for each sampling, at *p* ≤ 0.05; ns: non-significant. Different uppercase letters indicate significant differences between the three sampling times (First stress, Recovery, and Second stress) for each treatment, at *p* ≤ 0.05; NS: non-significant. Vertical bars indicate standard error (n = 4).

Monovalent (Na^+^, Cl^−^, K^+^) and divalent (Ca^2+^) ion contents were determined in the roots and leaves of *L. angustebracteatum* plants after each of the three experimental phases (Figure 4).

During the first stress period, the root and leaf Na^+^ and Cl^−^ contents increased in parallel with the increase in substrate salinity, whereas water deficit had no significant effect on the concentration of these ions in either the roots or in the leaves. The ‘recovery’ phase was fully effective, reducing ion levels to control values without any significant difference between treatments. The second stress phase mimicked the effects of the first one, with a concentration-dependent increase in the root and leaf Na^+^ and Cl^−^ levels in the salt-treated plants and no change in response to water stress; the only quantitative difference detected was that the leaf Cl^−^ concentrations were lower in the presence of 1 M NaCl than after the first stress phase (Figure 4A,B).

The root K^+^ concentrations increased slightly but significantly in response to the 1 M NaCl and the water deficit treatments, whereas they did not change in the leaves. The recovery phase caused a drop to control levels in the leaf and root K^+^ contents in the salt-stressed plants, and no significant differences were observed between treatments. Similarly, the root and leaf K^+^ concentrations in salt-treated and water-stressed plants did not differ significantly from the control values after the second stress period, although they slightly increased in the roots of the salt-stressed plants, with respect to the values measured after the recovery period (Figure 4C).

The patterns of Ca^2+^ variation in response to the experimental treatments were qualitatively similar to those of Na^+^, although reaching absolute values about 10-fold lower, in molar terms. Thus, during the two stress periods, the root and leaf Ca^2+^ levels increased significantly in the salt-stressed plants but maintained the same values as the corresponding controls in the plants subjected to a water deficit. After the intermediate recovery period, in general, no differences were observed between the previously stressed plants and the control, either in the roots or in the leaves (Figure 4D). It should be mentioned that in all the treatments, and for the four measured ions, the average contents were consistently higher in the leaves than in the roots (Figure 4).

### 2.5. Osmolyte Contents

**Figure 5:**
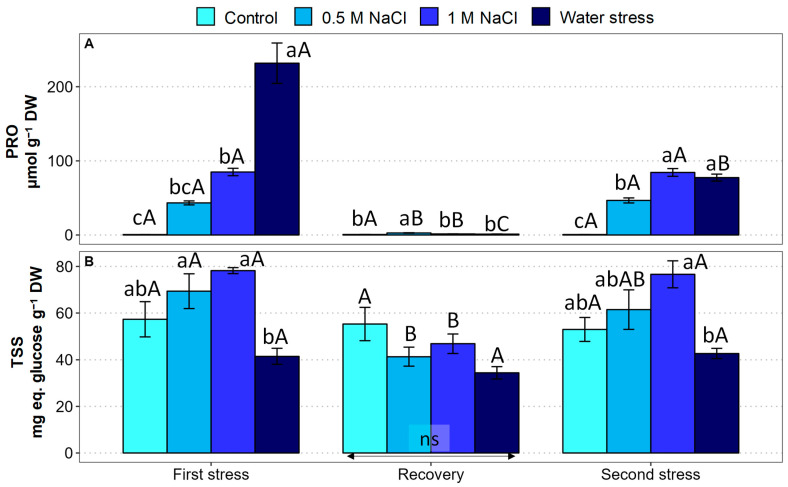
Effect of 30 days of stress treatment (First stress), followed by 15 days of watering with non-saline water (Recovery) and by additional 15 days of stress (Second stress) on the leaf concentrations of osmolytes in *Limonium angustebracteatum* plants. (**A**) Proline (PRO) and (**B**) Total Soluble Sugars (TSS). Different lowercase letters over the bars indicate significant differences between treatments (Control, 0.5 M NaCl, 1 M NaCl, and Water stress) for each sampling, at *p* ≤ 0.05; ns: non-significant. Different uppercase letters indicate significant differences between the three sampling times (First stress, Recovery, and Second stress) for each treatment, at *p* ≤ 0.05; NS: non-significant. Vertical bars indicate standard error (n = 4).

Different organic compounds have been reported as functional osmolytes in *Limonium* species, including some of the most common, e.g., proline and several sugars [24]. In the present work, the leaf contents of proline (PRO) and total soluble sugars (TSS) were determined in *L. angustebracteatum* plants subjected to the different stress treatments (Figure 5).

During the first stress period, mean PRO concentration increased substantially in response to the salt treatments, reaching ca. 85 µmol g^−1^ DW in the presence of 1 M NaCl, representing a 146-fold increase over the control. The increase in PRO levels was even stronger in plants subjected to the water deficit treatment, reaching almost 400-fold compared to the non-stressed control plants. Watering the pots with non-saline water during the subsequent recovery phase induced a drastic reduction in the PRO contents in salt and drought-treated plants to values close to the control and, in all cases, below 2 µmol g^−1^ DW. The second salt treatment had the same effect as the first one in terms of PRO accumulation; however, a milder response to water stress was observed, with the leaf PRO contents reaching approximately the same value as in the presence of 1 M NaCl, about 140-fold higher than in the controls (Figure 5A).

Mean TSS contents increased slightly in response to the first salt treatments and declined by about 37% under water stress; however, the differences with the control were not statistically significant in all cases. After recovery, the TSS contents were maintained in the control and the water-stressed plants, whereas in the salt-treated plants, they were almost halved so that no significant differences were detected between treatments. The second stress period produced a TSS accumulation pattern practically identical to the first one: the mean TSS contents increased in the salt-treated plants and decreased in those subjected to water stress, but, in all cases, the differences with the control plants were not statistically significant (Figure 5B).

### 2.6. Oxidative Stress Markers and Antioxidant Compounds

**Figure 6:**
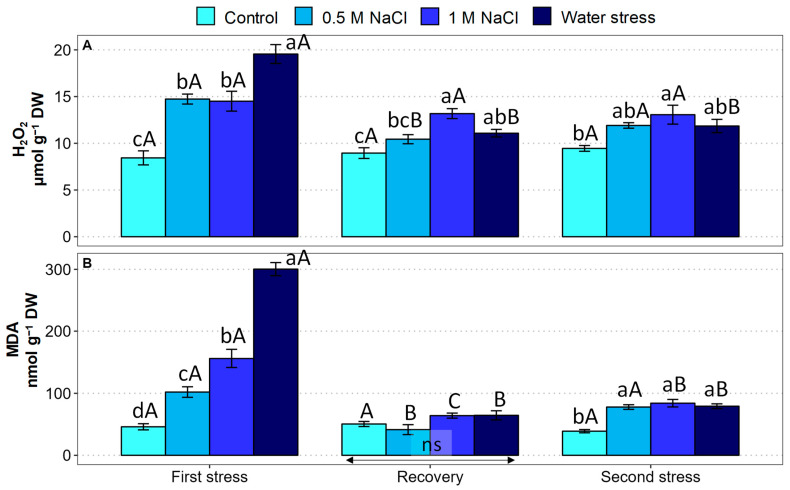
Effect of 30 days of stress treatment (First stress), followed by 15 days of watering with non-saline water (Recovery) and by additional 15 days of stress (Second stress) on leaf concentration of oxidative stress markers in *Limonium angustebracteatum* plants. (**A**) Hydrogen peroxide (H_2_O_2_) and (**B**) Malondialdehyde (MDA). Different lowercase letters over the bars indicate significant differences between treatments (Control, 0.5 M NaCl, 1 M NaCl, and Water stress) for each sampling, at *p* ≤ 0.05; ns: non-significant. Different uppercase letters indicate significant differences between the three sampling times (First stress, Recovery, and Second stress) for each treatment, at *p* ≤ 0.05; ns: non-significant. Vertical bars indicate standard error (n = 4).

To evaluate the potential generation of secondary oxidative stress in the *Limonium* plants exposed to salt or water stress treatments, the foliar contents of two reliable biochemical markers, hydrogen peroxide (H_2_O_2_) and malondialdehyde (MDA), were determined (Figure 6).

The H_2_O_2_ content increased significantly in the plants subjected to salinity, with no difference between the two NaCl concentrations, and somewhat more (about 2.3-fold) in the water-stressed plants. The recovery interval prompted a reduction in the H_2_O_2_ contents in the stressed plants, especially in the water-stressed ones; nevertheless, the values measured in the plants watered with 1 M NaCl and those previously subjected to water deficit remained significantly higher than the control. This pattern was essentially maintained after the second stress period (Figure 6A).

The changes observed in MDA concentrations were qualitatively similar to those of H_2_O_2_. The MDA contents increased significantly in parallel to increasing salinity during the first stress period, and even more, about 6.5-fold, in response to the drought treatment. During recovery, MDA levels dropped to control values in all stressed plants, so that no differences could be observed between the different treatments. In the second stress period, the MDA contents again increased slightly, but significantly, in all the stressed plants, compared with the non-stressed controls; however, the final absolute MDA concentrations measured in the water-stressed plants and those watered with 1 M NaCl were lower than the corresponding values determined after the first stress period (Figure 6B).

**Figure 7:**
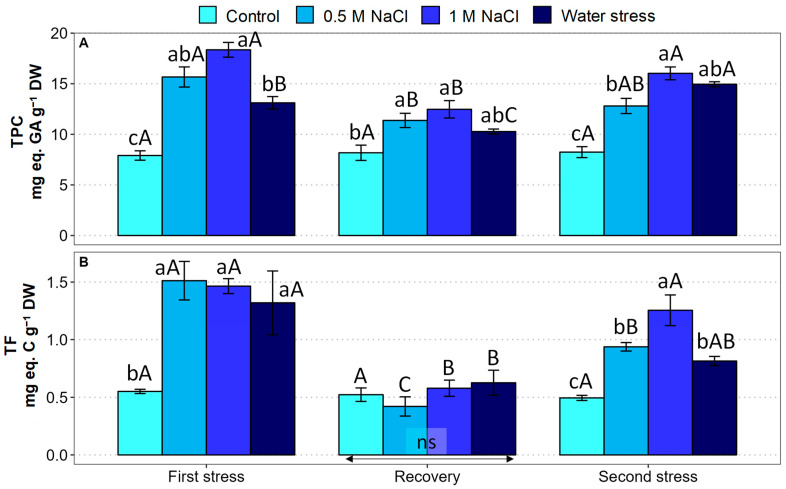
Effect of 30 days of stress treatment (First stress), followed by 15 days of watering with non-saline water (Recovery) and by additional 15 days of stress (Second stress) on leaf concentration of antioxidant compounds in *Limonium angustebracteatum* plants. (**A**) Total phenolic compounds (TPC) and (**B**) total flavonoids (TF). Different lowercase letters over the bars indicate significant differences between treatments (Control, 0.5 M NaCl, 1 M NaCl, and Water stress) for each sampling, at *p* ≤ 0.05; ns: non-significant. Different uppercase letters indicate significant differences between the three sampling times (First stress, Recovery, and Second stress) for each treatment, at *p* ≤ 0.05; ns: non-significant. Vertical bars indicate standard error (n = 4).

As expected, the generation of oxidative stress under conditions of salinity and water stress induced the synthesis of common antioxidant metabolites, phenolic compounds (TPC), including the subgroup of flavonoids (TF) (Figure 7).

During the first stress phase, significant increases in TPC (Figure 7A) and TF (Figure 7B) contents were observed. In the case of TPC, the relative increase over the control values varied between 1.7-fold for the water-stressed plants and 2.3-fold for the plants treated with 1 M NaCl. On the other hand, the relative increase in the TF concentration amounted to ca. 2-fold, without significant differences between the salt and water deficit treatments. After the recovery period, the TPC and TF concentrations decreased significantly in stressed plants; however, the TPC contents were still significantly higher than the control values in salt-treated plants (Figure 7A), whereas no differences between the treatments were observed for TF (Figure 7B). During the second stress phase, the TPC and TF contents of the salt and water-stressed plants increased again to values significantly higher than the control, reaching concentrations similar to those determined after the first stress period (Figure 7).

### 2.7. General Pattern of Trait Responses

Two Principal Component Analyses (PCAs) were performed to synthesise, with a multivariate approach, the relationships between the main measured traits during the first (PCAstress1) and the second phase of stress (PCAstress2). This also allows, at a glance, a visual comparison of the changes in the trait correlations before and after the recovery stage.

**Figure 8:**
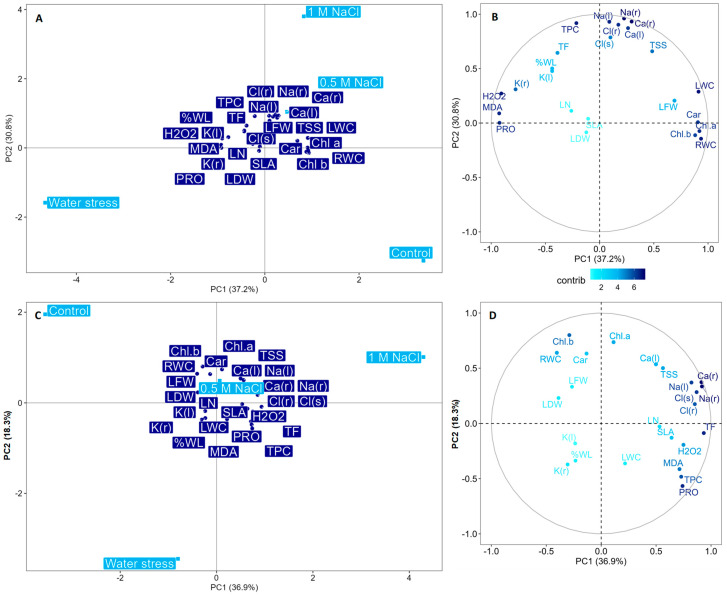
PCA biplot of variables (**A**) following the first stress phase (PCAstress1) and (**C**) the second stress phase (PCAstress2). Light blue squares indicate the barycentres of the four tested treatments (Control, 0.5 M NaCl, 0.1 M NaCl, and Water stress), while dark blue circles indicate the barycentres of the quantitative variables, i.e., the main measured variables, total leaf number (LN), wilted leaf percentage (%WL), specific leaf area (SLA), leaf fresh weight (LFW), leaf dry weight (LDW), leaf water content (LWC), root water content (RWC), chlorophyll a (Chl a), chlorophyll b (Chl b), carotenoids (Car), root sodium concentration (Na(r), leaf sodium concentration (Na(l)), root chloride concentration (Cl(r)), leaf chloride concentration (Cl(l)), root potassium concentration (K(r)), leaf potassium concentration (K(l)), root calcium concentration (Ca(r)), leaf calcium concentration (Ca(l)), proline (PRO), total soluble sugars (TSS), malondialdehyde (MDA), hydrogen peroxide (H_2_O_2_), total phenolic compounds (TPC), and total flavonoids (TF). Correlation circle plot of the main measured traits (**B**) following the first stress phase (PCAstress1) and (**D**) the second stress phase (PCAstress2). The growing colour tone of the dots, from light blue to dark blue, indicates the increasing contribution of a variable to the definition of the first two principal components.

The first three principal components (PCs) described 78.0% and 66.3% of the data variance in PCAstress1 and PCAstress2, respectively, and were selected for PCA analysis. In Figure 8, the biplots (Figure 8A,C) and the correlation circles (Figure 8B,D) of the first two components are shown, with the barycentres of the treatments and the 24 measured variables. The *p*-values of the correlation coefficients between the variables and each PC are shown in Table S2 in the Supplementary Materials.

In PCAstress1, PC1 mainly represented the effects of the water stress treatment (WS). The WS barycentre was located on the negative side of the PC1-axis (Figure 8A), indicating that it is negatively correlated with this component. As the PC1 results positively correlated with root water content, chlorophyll a, leaf water content, carotenoids, chlorophyll b, and leaf fresh weight (Figure 8B), we can sum up that the increase in the water stress caused a decrease in pigments, leaves, and root water content. On the other hand, the PC1 results negatively correlated with malondialdehyde, proline, hydrogen peroxide, and root K concentration (Figure 8B), meaning that the water deficit condition was counterbalanced by an increase in the accumulation of proline and potassium as the main osmoregulatory compounds, which were, however, not sufficient to prevent the generation of oxidative stress.

PC2, instead, mainly described the 0.1 M salt stress treatment as being significantly and positively correlated with PC2 and hence located on the positive side of the PC2-axis (Figure 8A). PC2, in turn, resulted in a strong positive correlation with root Na, root Ca, leaf Na, total phenolic compounds, root Cl, leaf Ca, leaf Cl, total soluble sugars, and total flavonoids (Figure 8B). This suggests that the accumulation of ions in both the below- and above-ground organs and the synthesis of antioxidant compounds such as phenols and flavonoids are the main mechanisms used by the *Limonium* plants to cope with the highest level of salinity. The third component resulted in a positive correlation with the specific leaf area, the leaf number, and the wilted leaf percentage (data not shown). However, as this component did not show a significant correlation with any of the four applied treatments, it emerges that these biometric parameters are not very responsive to the effects of water and salt stress.

In PCAstress2, there was an apparent switch in the position of the water stress and control treatments, whose barycentres appeared to be closer than in the first stress phase, whereas the barycentres of the two salinity stress treatments remained in the same position as in PCAStress1, in the upper-right quadrant (Figure 8C).

In PCAstress2, the first PC mainly referred to the response of the *Limonium* plants to the highest level of salinity. Indeed, PC1 resulted in a positive correlation with total flavonoids, root Na, root Ca, leaf Cl, root Cl, leaf Na, hydrogen peroxide, proline, total phenolic compounds, malondialdehyde, specific leaf area, total soluble sugars, number of leaves, and leaf Ca concentration. All these traits increased again when the salinity stress was reimposed but to a greater extent under the highest salinity level, whereas in the intermediate salinity level, the effect of the stress was generally weaker.

The second axis principally represented the *Limonium* plant’s response to water stress. The PC2 was positively correlated with the control treatment, whose barycentre was located on the positive PC2 axis, and negatively correlated with the water stress treatment, whose barycentre was located on the negative side of the PC2 axis. Indeed, the PC2 showed a positive correlation with chlorophyll b, chlorophyll a, root water content, carotenoids, leaf Ca, and total soluble sugars, and a negative correlation with proline. This indicated once again that the pigment contents were reduced under water deficit, although to a lower extent than during the first stress phase, and that the plants responded to the stress primarily by producing proline as an osmotic and antioxidant compound. It is worth noting that in this second stress phase, there were no significant correlations between PC2 and the oxidative stress markers, indicating a milder effect of water stress after the recovery stage.

The third component was negatively correlated with the number of leaves and the specific leaf area (data not shown) but, once again, this PC did not show a significant correlation with any of the four applied treatments. Again, this demonstrates that the measurement of these biometric parameters represents a not very informative analysis of the plant’s response to salinity and drought.

## 3. Discussion

Unravelling the role of stress memory in improving plant responses to future stress events is gaining more attention in plant stress research. The mechanisms underlying stress memory, however, are still not fully understood. In saline coastal environments, halophyte plants are naturally adapted to seasonal fluctuations or unpredictable changes in salinity and other co-occurring constraints such as drought and flooding [33,34]; hence, they may serve as a model to study plant stress memory. *Limonium angustebracteatum* is a threatened endemic halophyte species of the Iberian Peninsula with a high conservation interest. In the present study, *L. angustebracteatum* plants were exposed to two episodes of salinity and drought stress, separated by a recovery period.

*L. angustebracteatum* leaf fresh weight and water content did not change under 0.5 and 1 M NaCl salinity, similar to what was observed by Mir et al. [35], but were remarkably affected by water deficit, indicating that the species is highly salt tolerant but sensitive to drought. Since leaf dry weight did not vary after the salt or water stress treatments, it can be concluded that the drought-induced decrease in FW was primarily due to leaf dehydration and not to a reduction in biomass accumulation. In general, the ability of *Limonium* plants to maintain or even increase their fresh weight under salinity was already noted by many authors, with differences depending on the species [24]. In most cases, however, a FW reduction was generally observed above 200 mM NaCl salt concentration [19,21,36,37]. More controversial is the characterisation of this genus under drought stress. Indeed, similarly to our species, a halving of the fresh weight was recorded in *L. virgatum* under severe water deficiency [8], in contrast to the mild growth inhibition observed in *L. santapolense*, *L. virgatum*, *L. girardianum* and *L. narbonense* [25], and to the high water stress tolerance shown by *L. linifolium*, [38]. In any case, *L. angustebracteatum* plants exposed to drought in the first stress period could completely rehydrate their leaves during the recovery period and maintain leaf fresh and dry weights not significantly different from the control after the second drought stress phase.

Instead, the root water content was sensitive to salinity and decreased the most under water stress. This is plausible because the roots are the organ directly exposed to salt or drying soils and may act as a buffer to preserve the above-ground biomass homeostasis and growth [39]. However, the *L. angustebracteatum* plants re-established their root water content during the recovery, and the plants under 0.5 M NaCl salinity showed an increased ability to maintain their water homeostasis after the second stress exposition. This is likely related to an increased accumulation of osmolytes, such as soluble sugars and amino acids, as well as to thicker cell walls with higher lignin contents [40] through an induced molecular reprogramming for memory retention against salt stress [41].

A signal of greater adaptation to stress reiteration was also found in the number of wilted leaves, which did not increase during the second stress event. However, the stress imprint was insufficient to prevent the second drop in pigment content in the water-stressed plants, although the reduction was lighter compared to the first stress phase.

The decline in pigment concentration under drought stress may derive from lower chlorophyll synthesis, as an acclimation strategy to reduce the possibility of photodamage because of an excess of light energy not sufficiently dissipated under decreasing transpiration [42], as well as from the generation of reactive oxygen species damaging proteins, membrane lipids, and other components of the photosystems [43,44]. The higher pigment maintenance after the first stress experience might be related to a greater capacity to control the energy-dependent quenching and, ultimately, favour the dissipation of light energy to prevent photodamage [45,46,47].

As a recretohalophyte, the ability of the *L. angustebracteatum* to cope with a salinity level approximately twice as high as seawater is strongly based on its capacity to accumulate Na^+^ and Cl^−^ ions for osmotic adjustment and to excrete the excess salts through specialised salt glands located on the leaves [35]. The active accumulation of ions from the soil solution is more energetically favourable than the generation ex novo of organic osmolytes. Indeed, Na^+^ and Cl^−^ ions accumulation was observed during the first and second stress periods, without compromising the adsorption of K^+^ and Ca^2+^ that, especially for Ca^2+^, was evenly increased with salinity.

An increase in Ca^2+^ uptake with salinity was already observed in the Salicornioideae subfamily [48] and other halophytic species [49,50]. In fact, Ca^2+^ is a multifunctional player in the complex salt stress response networks, acting in membrane stability and exocytosis of toxic ions, as well as in the stress signal transduction pathways [51,52]. Knight et al. [53] suggested that the osmotic stress-induced Ca^2+^ response encodes a ‘memory’ of previously encountered stress and thus can facilitate plant acclimation to recurring environmental stresses. This matches the lower accumulation of calcium in the saline treatments during the second stress phase.

Interestingly, the lack of a further K^+^ increase in the drought treatment during the second stress, despite this element’s general use for osmoregulation and oxidative defence purposes, may be another signal of improved plant ability to withstand the lack of water.

It is worth noting that, after the salinity release, the concentration of Na^+^ and Cl^−^ ions in the leaves and roots of *Limonium* plants decreased to levels comparable to the control. This behaviour differed from what we previously observed in three Salicornioideae species, *Sarcocornia fruticosa*, *Salicornia europaea* and *Salicornia veneta* [48], in which the content of these ions remained unchanged after salt stress recovery, both in the roots and shoots, probably contributing to constitutive defence mechanisms against future osmotic imbalances, together with the osmoprotectant glycine betaine. In the *Limonium* plants, on the other hand, toxic Na^+^ and Cl^−^ ions are most likely excreted through the salt glands during the recovery treatment.

Along with the active uptake of ions, in the stressed plants there was an increased synthesis of proline, a compatible osmolyte whose accumulation is a well-documented response under several adverse conditions [54,55]. During the first stress phase, the proline increase was considerably higher under water scarcity than under salinity. In both cases, proline decreased to very low control levels during the recovery period, and increased again, but to a lesser extent than in the first stress period, during the second stress phase. This finding is in line with a previous report [56] and matches molecular data [57,58], demonstrating the role of proline in evoking drought memory and promoting the maintenance of the plant water status under recurrent drought events. Contrary to our data, other reports [59,60] showed higher proline levels in quinoa and tomato salt-injured plants after the recovery period. These authors considered the maintenance of higher levels of this osmolyte as a constitutive response mechanism for increasing stress resistance, as described above for glycine betaine in Salicornioideae species [48].

Conversely to proline, a moderate increase in soluble sugars was observed only in response to salt stress, whereas their content always remained below control levels in the water-stressed plants. Since sugars are constituent elements of the energy metabolism of plants, it is not easy to distinguish their specific effects in the response to environmental stress. However, sugars are involved in many mechanisms linked to stress memory, as they regulate the production of mRNA, interact with phytohormones, and control protein activity and stability [61]. These mechanisms, however, did not avoid a further significative sugar increase upon treatment with 1 M NaCl during the second stress period.

Malonaldehyde and hydrogen peroxide are commonly used to estimate the extent of peroxidation of membrane lipids under adverse conditions. In this study, these two side-products increased with increasing salinity and peaked under drought-stress conditions. However, their generation during the second stress episode was more contained, especially in the water-stressed plants and those watered with 0.5 M NaCl, which might indicate the possibility of acquired stress acclimation, similar to what was observed for the halophyte *Cakile maritima* [62]. Several studies involving reverse genetics have shown that increased endogenous H_2_O_2_ levels can confer further resistance to posterior oxidative stress impositions [63]. H_2_O_2_ is, indeed, characterised by high diffusivity and long persistence and acts as a signalling molecule in stress transduction pathways. In fact, it has been demonstrated that exogenous application of H_2_O_2_ on seeds and plants can enhance abiotic stress tolerance by reducing lipid peroxidation and modulating ROS detoxification [64,65].

Earlier studies also demonstrated that plants previously exposed to stress showed a higher production of antioxidant compounds, such as phenolics, including the subgroup of flavonoids, helping plant cells to maintain ROS homeostasis and protect the photosynthetic apparatus, proteins, lipids, and nucleic acids [66,67]. However, in our case, the lower occurrence of oxidative damage indicators during the second stress phase, especially under 0.5 M NaCl and drought stress, can explain the lower production of the phenolic and flavonoid compounds, suggesting a likely improved capacity to face cyclic stress. This is also visible in the PCA of the second stress, in which the barycentres of the 0.5 M NaCl and water stress treatments are opposed to those of the oxidative stress indicators.

However, the reduced levels of oxidative stress after the second stress incidence may result from the increased activity of antioxidant enzymes such as superoxide dismutase, catalase, different peroxidases, or glutathione reductase, which were not determined in the present study. Furthermore, in this initial attempt to elucidate possible ‘stress memory’ mechanisms in *L. angustebracteatum*, we did not consider the effect of phytohormones such as abscisic acid, jasmonates, gibberellins, ethylene, or salicylic acid that, according to earlier reports, exert a protective role under reiterated stress conditions by reprogramming gene expression [68,69,70] towards optimised plant responses. Further research should address the issues mentioned above, as well as the plant responses at the root level. The root apparatus, being the plant organ most directly exposed to the two stress agents, should receive higher priority in future studies, to provide more clues to interpreting whole plant behaviour.

## 4. Materials and Methods

### 4.1. Plant Material

*Limonium angustebracteatum* plants were obtained from seeds provided by the Centre for Forestry Research and Experimentation (CIEF), Regional Government of Valencia. Seeds were germinated in standard Petri dishes in a climatic chamber under controlled conditions of 16 h light/8 h dark at 25 °C. Seedlings were individually placed in 12 cm-diameter pots filled with a mixture of commercial peat and vermiculite (3:1), watered regularly with tap water, and transferred to a greenhouse with natural illumination, 65% relative humidity and a 23–30 °C temperature range.

### 4.2. Experimental Design

The experiment was carried out on one-year-old plants, from 25 May to 22 July 2021. Four treatments were applied: a control with freshwater irrigation, two salt stress treatments, irrigating the plants with 0.5 and 1 M NaCl solutions, respectively, and a water stress treatment by completely withholding irrigation.

The first stress phase, which lasted 30 days, was applied to 12 plants per treatment for a total number of 48 starting plants. During this phase, the 12 pots of each treatment were placed inside trays and were watered–except those subjected to the water stress treatment–filling the trays from the bottom with a volume of 0.13 L of water or NaCl solution per pot. After this period, four plants per treatment were harvested, and the remaining ones were allowed to recover through abundant pot washings with fresh water in the salt stress treatments (0.3 L pot^−1^) or by restoring the soil water content up to 80% in the water-stressed plants (0.25 L pot^−1^). In the two salt stress treatments, the percolation water was always removed from the trays to discharge the leached salts. After the recovery period, another four plants per treatment were harvested. The remaining four plants per treatment were subjected to a second stress, as in the first stress period: watering with fresh water for the control, irrigation with 0.5 M and 1 M NaCl solutions for the salt stress treatment, and complete withholding of irrigation for the water stress treatment, with four plants per treatment. The second stress phase was extended for 15 days, up to the onset of visual wilting symptoms in the water-stressed plants, after which all plants were harvested.

**Figure 9:**
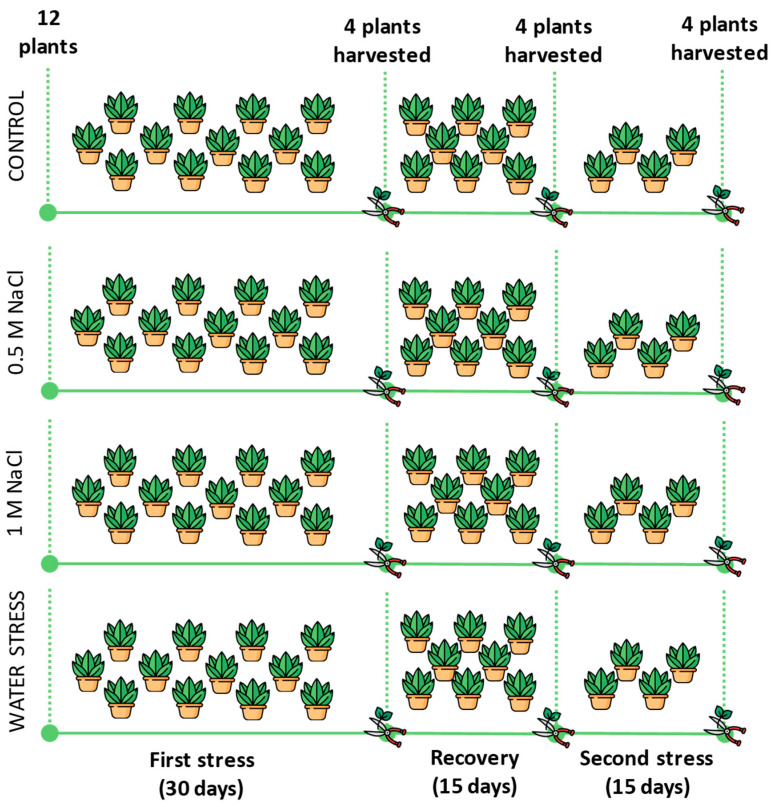
Scheme of the experimental phases: 12 plants were prepared for each of the four treatments (Control, 0.5 M NaCl, 1 M NaCl, Water stress). After 30 days of stress exposition (First stress), 4 plants per treatment were harvested, and the remaining 8 plants were allowed to recover for the next 15 days (Recovery). After that, another four plants were harvested and the remaining four were subjected to a further 15 days of stress before the final harvest.

The three stress phases will be named for simplification first stress, recovery, and second stress (Figure 9).

During the whole experiment, irrigation was administered twice per week, and the amount of water (L pot^−1^) distributed to each treatment during the three experimental stages is shown in Table 2.

### 4.3. Morphological Parameters

*Limonium angustebracteatum* above-ground biomass is composed of a dense leafy rosette. The following growth parameters were measured at each harvest time: total number of leaves, number of withered leaves, and leaf fresh weight (LFW). Three leaves per plant were randomly collected and scanned to calculate the mean leaf area. The total leaf area of the plants was estimated from the mean leaf area and the total number of leaves.

A fraction of the leaf fresh material was frozen in liquid N_2_ and stored at −75 °C. Another fraction was weighed (fresh weight, FW), dried in an oven at 65 °C for 48–72 h until constant weight, and weighed again (dry weight, DW). The relation between the FW and DW of this fraction was used to calculate the water content percentage (%) as follows:

(1) $$\mathrm{WC}\left(\%\right)=\frac{\mathrm{FW}-\mathrm{DW}}{\mathrm{FW}}\times 100$$

The proportion between the total leaf fresh weight (LFW) and the FW and DW of the oven-dried leaf fraction was used to estimate the total leaf dry weight (LDW).

The specific leaf area (SLA, cm^2^ g^−1^) was calculated dividing the total leaf area by the total leaf dry weight (LDW). A portion of the roots was collected from each pot, cleaned with a brush, weighed and oven-dried at 65 °C to determine the root water content as described above. An aliquot of the dry root material was then used for ion content determination, as explained below.

Pot substrate was sampled at each harvest phase for laboratory determination of water content and electrical conductivity (EC_1:5_). Substrate moisture was calculated gravimetrically with the method described above (Equation (1)), whereas the soil salinity was estimated by measuring the EC (dS m^−1^) of a 1:5 substrate to deionised water suspension, with a Crison 522 conductivity meter (Crison Instruments SA, Barcelona, Spain).

### 4.4. Photosynthetic Pigments

Fresh ground leaves (0.05 g) were used for measuring the concentration of chlorophyll a (Chl a), chlorophyll b (Chl b), and carotenoids (Car) by Lichtenthaler and Welburn’s classical method [71]. Extraction was performed with 1 mL ice-cold 80% acetone, and then the samples were placed in a shaker for 12 h in the dark at room temperature. Subsequently, they were centrifuged at 13,300× *g* for 10 min at 4 °C. The absorbance of the supernatants was measured at 470, 646 and 663 nm and the concentrations of the pigments were calculated according to the equations previously described [71].

### 4.5. Quantification of Ions

Concentrations of Na^+^, Cl^−^, K^+^, and Ca^2+,^ were determined separately in root and leaf material according to the protocol by Weimberg [72]. Samples of 0.1 g dry material were suspended in 2 mL of Milli-Q water, vortexed, and then mixed for 24 h in a shaker. Next, samples were incubated at 95 °C in a water bath for one hour, cooled on ice, and filtrated through a 0.45 µm nylon filter. The cations were quantified with a PFP7 flame photometer (Jenway Inc., Burlington, VT, USA), and Cl^−^ was measured using a chlorimeter Sherwood 926 (Cambridge, UK).

### 4.6. Quantification of Osmolytes

Proline (PRO) was quantified according to the protocol by Bates et al. [73]. PRO was extracted from 0.05 g fresh leaf material in 2 mL of 3% aqueous sulphosalicylic acid, mixed with acid ninhydrin solution, and incubated for one hour at 95 °C. After cooling on ice, two volumes of toluene were added to the sample and mixed by vortexing. The absorbance of the organic phase was read at 520 nm, using toluene as a blank. A standard curve was quantified in parallel samples containing known PRO concentrations. Finally, the PRO concentration was expressed as μmol g^−1^ DW.

The classical method described by Dubois et al. [74] was followed to measure the concentration of total soluble sugars (TSS). Fresh leaf material (0.05 g) was ground in liquid N_2_ and extracted with 80% (*v*/*v*) methanol. Samples were mixed in a rocker shaker for 24 h and then centrifuged at 13,300× *g* for 10 min. The collected supernatants were diluted with water and supplemented with concentrated sulphuric acid and 5% phenol and incubated for 20 min at room temperature. The absorbance was measured at 490 nm, and the TSS concentrations were expressed as equivalents of glucose, used as the standard (mg eq. glucose g^−1^ DW).

### 4.7. Determination of Oxidative Stress Markers and Antioxidant Compounds

Malondialdehyde (MDA), total phenolic compounds (TPC), and total flavonoids (TF) were measured in extracts prepared in 80% (*v*/*v*) methanol from 0.05 g ground fresh leaf material. MDA quantification was performed according to the method described by Hodges et al. [75] with some modifications [76]. Extracts were mixed with 0.5% thiobarbituric acid (TBA) prepared in 20% trichloroacetic acid (TCA) or with 20% TCA without TBA for the controls, and then incubated at 95 °C for 20 min, cooled on ice and centrifuged at 13,300× *g* for 10 min at 4 °C. The absorbance of the supernatants was measured at 532 nm. The non-specific absorbance at 600 and 440 nm was subtracted, and the MDA concentrations, expressed as nmol g^−1^ DW, were calculated [76].

H_2_O_2_ was measured according to the method by Loreto and Velikova [77]. Fresh leaf material (0.05 g) was extracted with a 0.1% (*w*/*v*) trichloroacetic acid (TCA) solution, followed by centrifuging the extract. Next, the supernatant was mixed with one volume of 10 mM potassium phosphate buffer (pH 7) and two volumes of 1 M potassium iodide. The absorbance was determined at 390 nm, and the concentration was calculated against an H_2_O_2_ standard calibration curve and expressed as µmol g^−1^ DW.

TPCs were quantified by reaction with the Folin-Ciocalteu reagent, according to the method described by Blainski et al. [78]. Methanol extracts were mixed with the reagent and 15% (*w*/*v*) Na_2_CO_3_ and incubated for 90 min in the dark. The absorbance was measured at 765 nm. A standard reaction was performed in parallel using known amounts of gallic acid (GA), and TPC contents were reported as equivalents of GA (mg eq. GA g^−1^ DW).

TFs were measured following the protocol by Zhisen et al. [79]. Methanol extracts were mixed with NaNO_2_ and AlCl_3_ under basic pH. Subsequently, after the reaction, the absorbance of the samples was determined at 510 nm. The concentration of TFs was expressed as equivalents of catechin, used as the standard (mg eq. C g^−1^ DW).

### 4.8. Statistical Analysis

The two experimental factors, i.e., the stress treatments (ST, four levels) and harvesting time (HT, three levels), were cross-combined, obtaining 12 treatments, each arranged with four completely randomised replicates, totalling 48 pots. This number of replicates is commonly adopted in experiments testing plant stress response in a controlled environment [80,81,82].

A two-way ANOVA was computed to test the sources of variation and estimate the interaction between the stress treatments (ST) and the harvesting times (HT), reporting the results in Table S1 in the Supplementary Materials.

Then, the collected data were analysed by two separated one-way ANOVAs for the respective four stress treatments (ST) and the three harvesting times (HT). Individual means were separated using Tukey’s honestly significant difference post hoc test at *p* < 0.05.

To identify possible groupings amongst all the dependent variables analysed at the end of the first and second stress phases and define the most important variables to distinguish the different treatments, the data were explored through two principal component analyses, denominated PCAstress1 and PCAstress2.

The principal components (PCs) were extrapolated from centred and scaled quantitative variables after the diagonalisation of the covariance matrix and the eigenvalue–eigenvector decomposition. The 24 measured traits were used as active quantitative variables, whereas the three harvest times and the four treatments (Control, 0.5 M NaCl, 1 M NaCl, and Water stress) were set as supplementary categorical variables, i.e., variables that are not projected along the principal component vectors. The *p*-values of the Pearson correlation coefficients between the quantitative variable and each PC were used to classify the variables according to their relevance (Table S2 in the Supplementary Materials). The eigenanalysis is displayed in Figure S1 in the Supplementary Materials.

All the analyses were performed with the R software environment (R Core Team, 2017), using the functions in the Car [83] and Emmeans [84] packages for the analysis of variance and post hoc tests and the FactoMineR [85] package for principal component analysis, while graphical representations relied on the ggplot2 [86] package.

## 5. Conclusions

*Limonium angustebracteatum* is an endemic halophyte from the Mediterranean Spanish coast that could be a suitable model to investigate the possibility of a ‘stress memory’ in the responses to seasonal changes in environmental stress conditions affecting salt marsh vegetation. Plants of *L. angustebracteatum* were subjected to controlled salt stress and water deficit treatments, followed by a recovery period and a second stress application, to assess their responses in terms of growth and changes in the levels of biochemical stress markers after each successive experimental phase.

The plants showed remarkable tolerance to salt stress, primarily based on the accumulation in leaves of high concentrations of Na^+^ and Cl^−^—together with the osmolyte proline—for osmotic adjustment, without affecting K^+^ uptake and transport. Ca^2+^, a well-known signalling molecule in stress response pathways, also increased in the roots and leaves with increasing salinity. The salt treatments generated oxidative stress, reflected by the increase in the contents of oxidative stress markers, MAD and H_2_O_2_; consequently, the accumulation of antioxidant compounds (phenolics and flavonoids) was also detected in salt-treated plants. The determination of growth parameters and photosynthetic pigments indicated that *L. angustebracteatum* is relatively sensitive to drought, particularly to water stress-induced dehydration of the plants. The water deficit treatment did not alter the ion patterns in the roots or leaves but induced the accumulation of proline and oxidative stress markers to higher concentrations than the salt stress treatment did.

All analysed variables were restored to control values, or at least decreased to values close to the corresponding controls, during the recovery period. The responses to the second stress mimicked those observed after the first stress period but were generally weaker, particularly in the case of water deficit, suggesting the occurrence of stress acclimation, or ‘stress memory’, induced by the application of the previous stress treatments.

The results described here will help design and implement conservation, regeneration, and reintroduction programmes for this threatened endemic halophyte. When selecting suitable zones for the growth of this species in salt marshes, soil salinity should not be a problem since *L. angustebracteatum* is highly salt tolerant and, thanks to the ‘stress memory’ effect, seems to be well adapted to extreme seasonal variations in salinity; it will also withstand the expected increase in salinity in these natural habitats due to climate change. However, areas prone to prolonged droughts should be avoided as the species is not so resistant to water deficit.

## Figures and Tables

**Table 1:** Total leaf number (LN), wilted leaf percentage (%WL), and specific leaf area (SLA) of *Limonium angustebracteatum* plants, measured at the beginning of the experiment (T0), and after 30 days of stress treatments (T30), 15 days of watering with non-saline water (T45), and another 15 days of stress (T60). Different lowercase letters indicate significant differences between treatments (Control, 0.5 M NaCl, 1 M NaCl, and Water stress) for each sampling time, at *p* ≤ 0.05. Different uppercase letters indicate significant differences between the sampling times (T0, First stress, Recovery, and Second stress) for each treatment, at *p* ≤ 0.05. Data are shown as mean ± standard error (n = 4).

	T0	First Stress (T30)	Recovery (T45)	Second Stress (T60)
Control				
total leaf no.	14 ± 1.20 aA	17 ± 1.40 aA	19 ± 1.96 aA	19 ± 1.64 aA
wilted leaf %	0 C	13 ± 2.70 bB	22 ± 2.68 aA	21 ± 2.39 abAB
SLA (cm^2^ g^−1^)		20.78 ± 3.59 aA	27.11 ± 3.08 aA	25.12 ± 5.59 aA
0.5 M NaCl				
total leaf no.	14 ± 1.21 aA	19 ± 2.09 aA	21 ± 3.30 aA	20 ± 3.43 aA
wilted leaf %	0 B	32 ± 6.80 aA	22 ± 2.94 aB	26 ± 2.67 aA
SLA (cm^2^ g^−1^)		37.14 ± 13.97 aA	25.46 ± 4.38 aA	31.17 ± 7.66 aA
1 M NaCl				
total leaf no.	13 ± 0.96 aB	16 ± 0.97 aA	15 ± 1.32 aA	28 ± 7.73 aA
wilted leaf %	0 C	41 ± 1.92 aA	38 ± 1.70 abA	19 ± 1.65 bA
SLA (cm^2^ g^−1^)		16.90 ± 3.15 aA	25.37 ± 4.14 aA	52.89 ± 18.08 aA
Water Stress				
total leaf no.	13 ± 1.02 Aa	16 ± 0.83 aA	16 ± 2.27 aA	18 ± 2.84 aA
wilted leaf %	0 B	38 ± 4.88 aA	50 ± 12.21 aA	26 ± 2.97 aA
SLA (cm^2^ g^−1^)		25.48 ± 8.21 aA	39.56 ± 9.43 aA	36.90 ± 4.60 aA

**Table 2:** Total amount of water (L pot^−1^) distributed during the two stress periods and the recovery phase in the four treatments.

	Control	0.5 M NaCl	1 M NaCl	Water Stress
First stress—30 days	1.17	1.17	1.17	0
Recovery—15 days	0.65	1.5	1.5	1.25
Second stress—15 days	0.65	0.65	0.65	0
Total (L pot^−1^)	2.47	3.32	3.32	1.25

## Data Availability

Data is contained within the article or supplementary material.

## Supplementary Materials

The following supporting information can be downloaded at: https://www.mdpi.com/article/10.3390/plants12010191/s1. Table S1: Two-way analysis of variance (ANOVA) of stress treatments (Treat), harvesting time (Time), and their interactions (Treat x Time) for the 24 measured traits: total leaf number (LN), wilted leaf percentage (%WL), specific leaf area (SLA), leaf fresh weight (LFW), leaf dry weight (LDW), leaf water content (LWC), root water content (RWC), proline (PRO), total soluble sugars (TSS), malondialdehyde (MDA), hydrogen peroxide (H_2_O_2_), total phenolic compounds (TPC), total flavonoids (TF), chlorophyll a (Chl a), chlorophyll b (Chl b), carotenoids (Car), root sodium concentration (Na(r)), leaf sodium concentration (Na(l)), root chloride concentration (Cl(r)), leaf chloride concentration (Cl(l)), root potassium concentration (K(r)), leaf potassium concentration (K(l)), root calcium concentration (Ca(r)), leaf calcium concentration (Ca(l)). Significance codes: ^ns^, ^(+)^, *, **, and *** mean, respectively, not significant and significant at *p* ≤ 0.1, *p* ≤0.05, *p* ≤ 0.01 and *p* ≤ 0.001. Table S2: Correlation coefficients between the first three PCs and the 24 measured variables: total leaf number (LN), wilted leaf percentage (%WL), specific leaf area (SLA), leaf fresh weight (LFW), leaf dry weight (LDW), leaf water content (LWC), root water content (RWC), chlorophyll a (Chl a), chlorophyll b (Chl b), carotenoids (Car), root sodium concentration (Na(r)), leaf sodium concentration (Na(l)), root chloride concentration (Cl(r)), leaf chloride concentration (Cl(l)), root potassium concentration (K(r)), leaf potassium concentration (K(l)), root calcium concentration (Ca(r)), leaf calcium concentration (Ca(l)), proline (PRO), total soluble sugars (TSS), malondialdehyde (MDA), hydrogen peroxide (H_2_O_2_), total phenolic compounds (TPC), and total flavonoids (TF). The correlations coefficients are accompanied by their relative *p* values. Figure S1: Eigen analysis of PCAstress1 and PCAstress2 correlation matrix.
