# Supplementary material for: Effect of Recurrent Salt and Drought Stress Treatments on the Endangered Halophyte Limonium angustebracteatum Erben
Source: Plants (Basel). 2023 Jan 3;12(1):191. doi: 10.3390/plants12010191 (PMC9823942; doi:10.3390/plants12010191)
Supplement: Supplementary file 1 [file plants-12-00191-s001.zip › plants-2091261-supplementary.pdf]

Table S1: Two-way analysis of variance (ANOVA) of stress treatments (Treat), harvesting time (Time), and their interactions (Treat x Time) for the 24 measured traits: total leaf number (LN), wilted leaf percentage (%WL), specific leaf area (SLA), leaf fresh weight (LFW), leaf dry weight (LDW), leaf water content (LWC), root water content (RWC), proline (PRO), total soluble sugars (TSS), malondialdehyde (MDA), hydrogen peroxide (H_2O_2), total phenolic compounds (TPC), total flavonoids (TF), chlorophyll a (Chl a), chlorophyll b (Chl b), carotenoids (Car), root sodium concentration (Na(r)), leaf sodium concentration (Na(l)), root chloride concentration (Cl(r)), leaf chloride concentration (Cl(l)), root potassium concentration (K(r)), leaf potassium concentration (K(l)), root calcium concentration (Ca(r)), leaf calcium concentration (Ca(l)). Significance codes: ^{ns}, $^{(+)}$, *, **, and *** mean, respectively, not significant and significant at p ≤ 0.1, p ≤0.05, p ≤ 0.01 and p ≤ 0.001

Treat	LN	WL	SLA	LFW	LDW	LWC	RWC	PRO	TSS	MDA	H_2O_2	TPC	TF	Chl a	Chl b	Car	Na(r)	Na(s)	Cl(r)	Cl(l)	K(r)	K(l)	Ca(r)	Ca(l)
**C**	17	12 c	24.34	5.16 ab	1.74	66.8 a	60.2 a	0.63 b	55.2 b	45.2 b	9.0	8.11 c	0.52 b	2.26 a	0.71 a	0.73	183.0 b	599.5 c	449.2 c	774 c	124.9 b	263.3	27.4 c	99.5 bc
**0.5 M NaCl**	18	18 b	31.26	5.24 a	1.76	66.3 a	53.5 b	30.9 a	57.4 ab	73.8 a	12.4	13.3 b	0.96 a	1.97 ab	0.51 b	0.66	517.5 ab	989.0 b	906.2 b	1203.3 b	141.6 ab	274.9	55.1 b	110.4 b
**1 M NaCl**	16	24 a	31.72	4.87 ab	1.51	69.1 a	49.0 b	56.9 a	67.2 a	101.4 a	13.6	15.6 a	1.10 a	2.12 a	0.57 ab	0.67	1027.2 a	1366.1 a	1526.4 a	2423.5 a	119.5 b	278.8	88.6 a	156.1 a
**WS**	15	27 a	33.98	3.88 b	1.72	52.4 b	42.1 c	103.4 a	39.5 c	148.0 a	14.2	12.8 b	0.92 a	1.54b	0.39 c	0.54	180.2 b	650.8 c	578.2 bc	810.5 c	163.9 a	293.5	25.9 c	77.4 c
p	0.314 ns	1.2e-06 ***	0.542 ns	0.030 *	0.690 ns	6.6e-08 ***	4.7e-09 ***	3.7e-06 ***	5.1e-06 ***	0.0001 **	1.4e-05 ***	7.9e-15 ***	3.4e-06 ***	0.002 **	8.1e-07 ***	0.313 ns	0.022 *	9.6e-12 ***	2.9e-09 ***	2.0e-5 ***	0.007 **	0.806 ns	7.2e-15 ***	1.3e-08 ***
**Time**																								
**T1**	17	31	25.08	4.46	1.84	53.7 b	38.2 c	90.1 a	61.6 a	151.1 a	14.3	13.8 a	1.21 a	1.58 b	0.48 b	0.56 b	657.5 a	997.8 a	1085.4 a	1782.1 a	173.3 a	325.4 a	59.9 a	131.2 a
**T2**	18	33	29.38	4.92	1.63	67.9 a	64.3 a	1.50 b	44.5 b	55.1 c	10.9	10.6 b	0.54 c	2.37 a	0.66 a	0.8 a	158.4 b	627.1 b	417.8 b	861.3 b	96.9 c	248.9 b	25.3 b	91.1 b
**T3**	21	23	36.52	4.98	1.58	69.4 a	51.1 b	52.3 a	58.4 a	70.0 b	11.6	13.0 a	0.88 b	1.97 b	0.49 b	0.59 b	615.1 a	1079.1 a	1091.7 a	1265.1 a	142.3 b	258.6 b	62.6 a	110.3 b
p	0.075 ns	0.110 ns	0.169 ns	0.405 ns	0.423 ns	3.1e-09 ***	1.8e-15 ***	0.009 **	0.0001 ***	0.0006 **	0.025 *	1.4e-07 ***	4.0e-09 ***	0.0001 ***	0.0001 ***	0.0008 ***	0.001 **	1.3e-07 ***	3.6e-07 ***	0.071 (+)	3.2e-07 ***	0.002 **	1.6e-10 ***	0.0001 ***
**Treat x Time**																								
**C-T1**	17	13 c	20.78	5.13	1.73	68.5 a	57.4 ab	0.58 f	57.3 abcd	46 f	8.44 g	7.91 f	0.55 d	2.49 ab	0.78 a	0.79 ab	204.6 bcd	498.4 d	455.0 d	627.3 e	119.9 bcd	260.7 bc	30.9 c	93.9 bc
**0.5M-T1**	19	32 b	37.14	5.02	1.93	62.3 a	42.5 cd	43.3 c	69.4 ab	102 bc	14.7 ab	15.7 abc	1.51 a	1.60 bcd	0.48 bc	0.56 de	817.5 ab	1040.2 bc	1305.9 bc	1405.3 ab	169 abc	340.3 ab	75.1 b	146.9 ab
**1M-T1**	16	41 a	16.90	5.11	1.70	66.5 a	37.3 d	85.0 b	78.2 a	156.2 ab	14.5 b	18.4 a	1.46 ab	1.72 abc	0.53 abc	0.63 bcde	1390.9 a	1807.5 a	2014.4 ab	4348.5 a	187.5 ab	357.4 a	109.0 a	199.4 a
**WS-T1**	16	38 a	25.48	2.58	1.99	17.7 b	15.7 e	231.7 a	41.5 cd	300.4 a	19.6 a	13.1 bcd	1.32 abc	0.53 d	0.15 d	0.24 f	216.9 bcd	645.0 cd	566.4 cd	747.3 de	217.1 a	343.3 ab	24.7 c	84.6 c
**C-T2**	19	22 bc	27.11	5.21	1.83	64.3 a	64.5 a	0.68 f	55.3 abcd	50.5 f	8.95 fg	8.18 ef	0.52 d	2.16 abc	0.67 ab	0.72 abcd	171.9 cd	534.4 d	441.7 d	776.0 de	125.1 bcd	262.9 bc	25.0 c	93.9 bc
**0.5M-T2**	21	22 bc	25.46	4.78	1.58	66.7 a	63.4 a	2.73 d	41.3 cd	41.4 f	10.4 ef	11.4 de	0.42 d	2.05abc	0.56 abc	0.8 a	148.3 d	589.7 cd	363.2 d	790.8 de	94.0 cd	239.7 c	21.6 c	83.3 c
**1M-T2**	15	38 a	25.37	4.90	1.39	71.8 a	62.4 a	1.41 e	46.9 bcd	64.0 e	13.2 bc	12.5 cd	0.58 d	2.48 ab	0.68 ab	0.77 abc	144.7 d	783.9 cd	389.5 d	1129.4 bc	56.3d	225.2 c	23.5 c	121.4 bc
**WS-T2**	16	50 a	39.56	4.78	1.73	68.8 a	66.9 a	1.18 e	34.4 d	64.5 e	11.1 de	10.3 def	0.63 d	2.78 a	0.72 ab	0.92 a	168.7 cd	600.3 cd	476.9 d	748.8 de	112.1 bcd	267.9 bc	31.2 c	65.8 c
**C-T3**	19	21 bc	25.12	5.14	1.66	67.6 a	58.6 ab	0.62 f	53 abcd	38.9 f	9.45 fg	8.2 ef	0.50 d	2.14 abc	0.69 ab	0.67 bcde	172.6 cd	765.7 cd	450.9 d	918.6 cde	129.9 bcd	266.3 bc	26.4 c	110.6 bc
**0.5M-T3**	20	26 bc	31.17	5.92	1.78	70.0 a	54.7 abc	46.6 c	61.5 abc	77.9 d	11.9 cd	12.8 bcd	0.94 bcd	2.27 abc	0.49 bc	0.62 bcde	586.6 bc	1337 ab	1049.5 cd	1413.6 b	162.0 abc	244.7 c	68.7 b	101.1 bc
**1M-T3**	28	19 bc	52.89	4.60	1.44	69.1 a	47.3 bcd	84.4 b	76.6 a	84.1 cd	13.1 bc	16.0 ab	1.25 abc	2.18 abc	0.49 bc	0.6 cde	1546.1 a	1506.8 ab	2175.3 a	1792.6 ab	114.7 bcd	253.9 c	133.3 a	147.7 ab
**WS-T3**	18	26 bc	36.90	4.27	1.45	70.9 a	43.9 cd	77.4 b	42.7 bcd	79.2 d	11.9 cde	14.9 bc	0.82 cd	1.3 cd	0.31 cd	0.45 ef	155.0 d	707.0 cd	691.2 cd	935.4 cd	162.5 abc	269.4 bc	21.9 c	81.9 c
p	0.165 ns	4.9e-06 ***	0.207 ns	0.257 ns	0.952 ns	1.7e-11 ***	9.7e-08 ***	4.3e-06 ***	0.04 *	1.6e-05 ***	4.2e-05 ***	0.0001 **	0.0012 **	0.0001 ***	8.4e-05 ***	0.0018 **	0.0008 ***	2.4e-05 ***	1.6e-05 ***	0.0003 ***	0.0048 **	0.050 *	1.4e-10 ***	0.019 *

Table S2: Correlation coefficients between the first three PCs and the 24 measured variables: total leaf number (LN), wilted leaf percentage (%WL), specific leaf area (SLA), leaf fresh weight (LFW), leaf dry weight (LDW), leaf water content (LWC), root water content (RWC), chlorophyll a (Chl a), chlorophyll b (Chl b), carotenoids (Car), root sodium concentration (Na(r)), leaf sodium concentration (Na(l)), root chloride concentration (Cl(r)), leaf chloride concentration (Cl(l)), root potassium concentration (K(r)), leaf potassium concentration (K(l)), root calcium concentration (Ca(r)), leaf calcium concentration (Ca(l)), proline (PRO), total soluble sugars (TSS), malondialdehyde (MDA), hydrogen peroxide (H_2O_2), total phenolic compounds (TPC), and total flavonoids (TF). The correlations coefficients are accompanied by their relative p values

	**PCAstress1**						**PCAstress2**					
	**PC1**		**PC2**		**PC3**		**PC1**		**PC2**		**PC3**	
	**R^2**	***p***	**R^2**	***p***	**R^2**	***p***	**R^2**	***p***	**R^2**	***p***	**R^2**	***p***
Quantitative variables												
**%WL**	-0.44	0.090	0.50	0.048	0.64	0.008	-0.23	0.383	-0.34	0.202	0.42	0.109
**Ca(l)**	0.26	0.327	0.87	1.05E-05	-0.10	0.723	0.50	0.049	0.54	0.032	-0.14	0.617
**Ca(r)**	0.30	0.267	0.93	1.43E-07	-0.04	0.889	0.91	8.11E-07	0.37	0.153	0.04	0.895
**Car**	0.91	1.28E-06	0.01	0.977	0.02	0.956	-0.13	0.623	0.63	0.009	0.43	0.094
**Chl.a**	0.92	4.29E-07	-0.07	0.785	0.06	0.827	0.11	0.677	0.74	0.001	0.40	0.129
**Chl.b**	0.88	6.46E-06	-0.11	0.680	0.12	0.661	-0.29	0.276	0.80	0.000	-0.41	0.119
**Cl(r)**	0.17	0.522	0.90	1.61E-06	-0.10	0.706	0.85	2.76E-05	0.18	0.515	0.08	0.761
**Cl(s)**	0.10	0.713	0.79	2.98E-04	-0.19	0.474	0.87	1.26E-05	0.28	0.285	-0.01	0.959
**H_2O_2**	-0.91	1.32E-06	0.27	0.309	-0.03	0.901	0.75	8.40E-04	-0.19	0.473	0.33	0.216
**K(l)**	-0.44	0.090	0.48	0.061	0.06	0.836	-0.24	0.378	-0.18	0.503	-0.02	0.928
**K(r)**	-0.77	4.22E-04	0.31	0.243	-0.23	0.401	-0.31	0.250	-0.37	0.157	0.49	0.055
**LDW**	-0.12	0.651	-0.09	0.753	-0.39	0.134	-0.39	0.137	0.23	0.389	0.55	0.026
**LFW**	0.69	3.07E-03	0.21	0.445	-0.24	0.375	-0.27	0.320	0.33	0.207	0.50	0.048
**LN**	-0.26	0.329	0.11	0.679	0.83	8.05E-05	0.53	0.034	-0.03	0.920	-0.52	0.037
**LWC**	0.91	8.41E-07	0.29	0.279	0.13	0.620	0.22	0.419	-0.36	0.169	-0.07	0.783
**MDA**	-0.93	2.65E-07	0.09	0.743	-0.17	0.519	0.71	0.002	-0.41	0.112	0.49	0.053
**Na(l)**	0.09	0.742	0.93	1.78E-07	-0.06	0.814	0.82	9.41E-05	0.37	0.157	0.10	0.717
**Na(r)**	0.23	0.401	0.96	3.36E-09	-0.02	0.930	0.92	5.71E-07	0.34	0.203	-0.03	0.912
**PRO**	-0.92	3.45E-07	0.00	0.994	-0.10	0.716	0.74	0.001	-0.57	0.022	0.14	0.597
**RWC**	0.94	1.03E-07	-0.14	0.595	0.15	0.569	-0.40	0.121	0.64	0.008	0.00	0.991
**SLA**	-0.11	0.697	0.04	0.884	0.93	1.19E-07	0.64	0.007	-0.13	0.635	-0.58	0.018
**TF**	-0.39	0.137	0.64	0.007	0.13	0.641	0.93	1.21E-07	-0.09	0.750	0.00	1.000
**TPC**	-0.21	0.425	0.92	5.33E-07	0.03	0.909	0.73	0.001	-0.48	0.059	0.27	0.319
**TSS**	0.48	0.057	0.66	0.005	-0.05	0.865	0.56	0.023	0.50	0.048	0.32	0.226
Categorical variables												
**trt=C**	3.37	0.006	-3.26	0.002967	-0.11	0.885391	-3.56	0.003	1.95	0.032	-1.10642	0.135
**trt=L**	0.47	0.738	1.04	0.410241	1.13	0.104169	0.07	0.963	0.48	0.622	1.373669	0.057
**trt=H**	0.83	0.554	3.80	0.000156	-0.75	0.295438	4.29	6.25E-05	1.01	0.298	-0.17172	0.824
**trt=WS**	-4.66	1.89E-06	-1.58	0.202734	-0.28	0.704006	-0.80	0.566	-3.45	2.08E-08	-0.09553	0.901

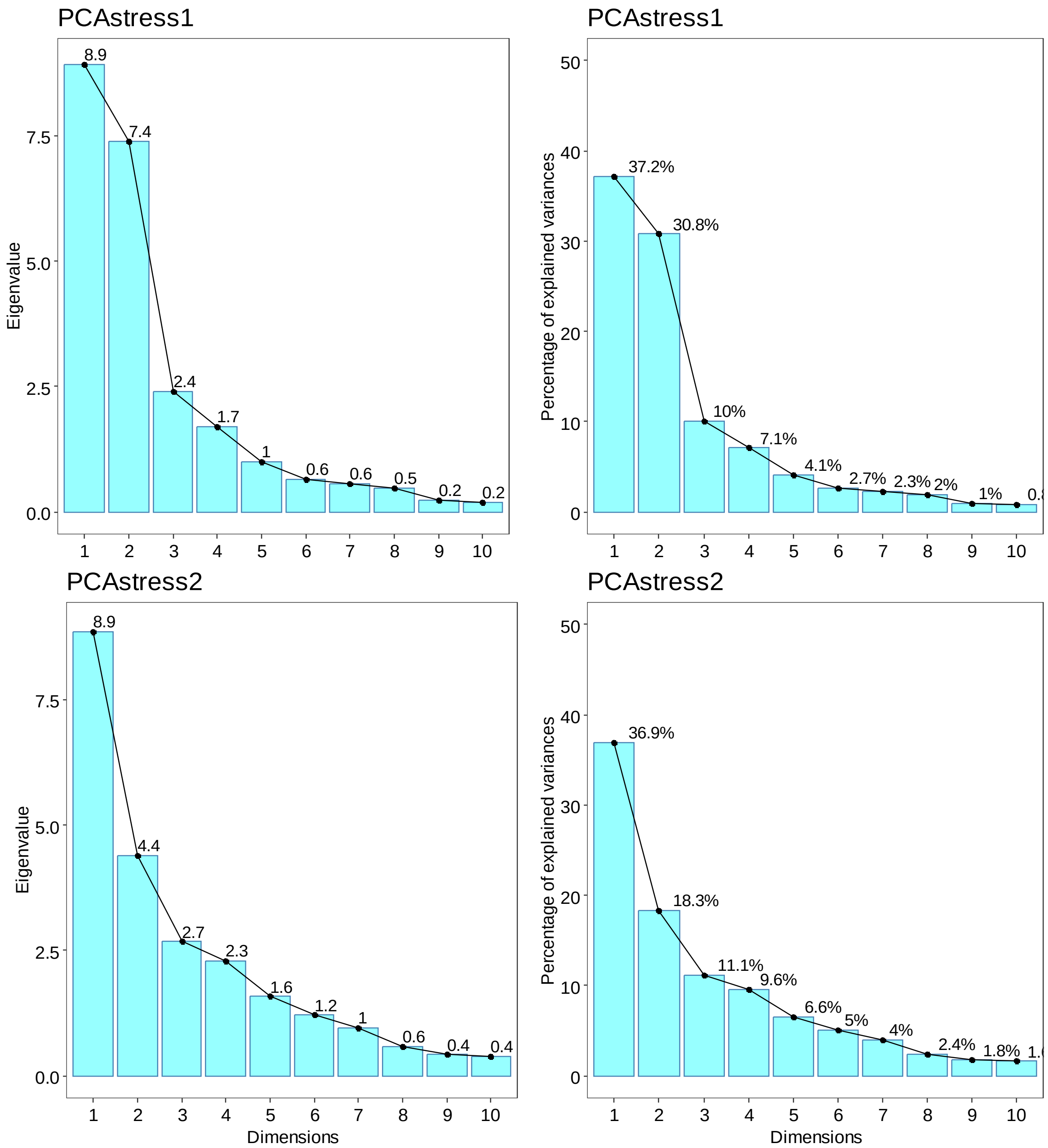

Figure S1: Eigen analysis of PCAstress1 and PCAstress2 correlation matrix
